# Antimicrobial combinations against *Helicobacter pylori* including benzoxadiazol-based flavodoxin inhibitors: *in vitro* characterization

**DOI:** 10.1128/spectrum.02623-23

**Published:** 2023-12-12

**Authors:** Lilha Beyria, Ophelie Gourbeyre, Sandra Salillas, Alejandro Mahía, María Dolores Díaz de Villegas, José Antonio Aínsa, Javier Sancho, Alain Bousquet-Mélou, Aude A. Ferran

**Affiliations:** 1 INTHERES, Université de Toulouse, INRAE, ENVT, Toulouse, France; 2 Biocomputation and Complex Systems Physics Institute (BIFI)-Joint Units: BIFI-IQFR (CSIC) and GBsC-CSIC, University of Zaragoza, Zaragoza, Spain; 3 Departamento de Bioquímica y Biología Molecular y Celular, Facultad de Ciencias, University of Zaragoza, Zaragoza, Spain; 4 Aragon Health Research Institute (IIS Aragón), Zaragoza, Spain; 5 CSIC—Departamento de Química Orgánica, Instituto de Síntesis Química y Catálisis Homogénea (ISQCH), University of Zaragoza, Zaragoza, Spain; 6 Departamento de Microbiología, Pediatría, Radiología y Salud Pública, Facultad de Medicina, University of Zaragoza, Zaragoza, Spain; 7 CIBER de Enfermedades Respiratorias–CIBERES, Instituto de Salud Carlos III, Madrid, Spain; Tainan Hospital, Ministry of Health and Welfare, Tainan, Taiwan

**Keywords:** *Helicobacter pylori*, checkerboard, time-kill studies, drug interactions, drug combination

## Abstract

Antimicrobial resistance of *Helicobacter pylori* (*Hp*) threatens currently available treatment regimens and prompts the development of antimicrobial drugs against new bacterial targets. One such class of drugs is *Hp*-flavodoxin (*Hp*-fld) inhibitors, which target an essential metabolic pathway in *Hp*. The aim of this study was to characterize the effects of these inhibitors against *Hp* over time when used alone or combined with conventional antibiotics. Four benzoxadiazol-based fld inhibitors were initially tested for their *in vitro* potency, with compound IV displaying the highest efficacy. This compound was then combined with clarithromycin, metronidazole, amoxicillin, levofloxacin, and rifampicin in further experiments. Checkerboard assays indicated that compound IV exhibited additive activity when combined with these antibiotics. The study further investigated the time course of antibacterial effects by exposing a high inoculum of *Hp* to compound IV and each antibiotic, alone or in combination. At just four times the minimum inhibitory concentration, compound IV demonstrated bactericidal effects within 6 h. However, the regrowth of bacteria occurred between 24 and 48 h of exposure. Similar patterns of killing followed by regrowth were observed for conventional antibiotics alone. However, the addition of compound IV to the antibiotics clearly limited regrowth over 72 h. These findings suggest that compound IV can effectively eliminate spontaneous mutants that are resistant to conventional antibiotics or prevent new mutations during drug exposure. *Hp*-fld inhibitors, such as compound IV, hold promise as new drugs to be integrated into *Hp* infection treatment, potentially reducing the development of resistance and shortening the duration of treatment.

**IMPORTANCE:**

The antimicrobial resistance of *Helicobacter pylori* (*Hp*) currently poses a threat to available treatment regimens. Developing antimicrobial drugs targeting new bacterial targets is crucial, and one such class of drugs includes *Hp*-flavodoxin (*Hp*-fld) inhibitors that target an essential metabolic pathway in *Hp*. Our study demonstrated that combining these new drugs with conventional antibiotics used for *Hp* infection treatment prevented the regrowth observed with drugs used alone. *Hp*-fld inhibitors show promise as new drugs to be incorporated into the treatment of *Hp* infection, potentially reducing the development of resistance and shortening the treatment duration.

## INTRODUCTION

*Helicobacter pylori* (*Hp*) is one of the most common bacterial pathogens in humans and is closely associated with gastrointestinal diseases such as peptic ulcer diseases, chronic gastritis, or gastric cancer. The pathogen infects more than half of the world’s population (1), and although most infected people are asymptomatic, *Hp* colonization of the gastric epithelial cells can cause an inflammatory response in the mucosa (2). The eradication of *Hp* has been recommended in order to decrease inflammation and prevent its progression to pre-neoplastic lesions, as well as the development of gastric cancer and other extragastric diseases (3, 4).

The standard triple therapies, recommended for the last two decades, include a proton pump inhibitor combined with two antibiotics (3). These therapies reached eradication rates greater than 80–90% in the 1990s, which decreased in recent years to rates as low as 60% (5). The high genetic diversity of *Hp* allows the bacterium to evade the immune response (6) and adapt to environmental challenges such as exposure to antimicrobials. One main explanation for the decrease in eradication rates is the rise of *Hp* resistance to antibiotics (3, 7). The resistance now reaches more than 15% of isolates all over the world, regarding clarithromycin, metronidazole, and levofloxacin (8); resistance to clarithromycin is even slightly above 20% in Europe (9). Since clarithromycin is the most effective and well-tolerated antibiotic used in the treatment of *Hp* infection, the development of resistance to this drug causes increased treatment failure, and the clarithromycin-resistant *Hp* strains were listed in 2017 as high-priority bacteria that threaten human health by the World Health Organization (WHO) (10, 11).

Current treatments are obviously under pressure due to the emergence of resistance, and ongoing efforts are made for the development of alternatives for the management of *Hp* infection, such as anti-virulence agents or antibacterial agents with new targets (12). These alternatives should result in higher eradication rates, provide good patient compliance, and prevent the increase in *Hp* antimicrobial resistance. Among the different strategies available, the development of drugs with specific targets for *Hp* seems promising, and key *Hp* gene products have been proposed for directed therapies (13 – 15). One of them is flavodoxin (*Hp*-fld), a small electron transfer protein involved in an essential *Hp* metabolic pathway that is absent in vertebrates (16, 17). The activity of this protein can be inhibited by derivatives of 7-nitrobenzoxadiazole (18). Those small molecules showed antibacterial properties with a low minimum inhibitory concentration (MIC) against several *Hp* strains, including those resistant to currently used antibiotics (19). The combination of these *Hp*-fld inhibitors with other antibiotics could potentially increase the eradication rate and lower the selection of resistance by killing bacteria that are resistant to currently used antibiotics. This study aimed first to analyze the type of interaction between *Hp*-fld inhibitors and other antibiotics on three *Hp* strains in standard inoculums. Subsequently, we sought to assess whether these combinations had the potential to effectively eliminate high bacterial inoculums, even when they may contain spontaneously resistant mutants to antibiotics.

## MATERIALS AND METHODS

### Antibiotics

**FIG 1:**
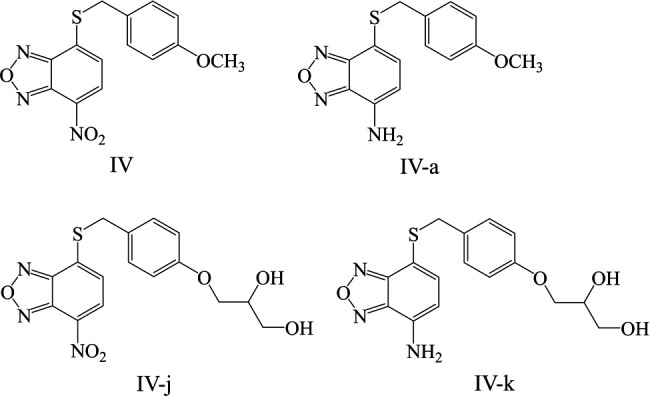
Chemical structures of lead compound IV (4-[(4-methoxyphenyl)methylsulfanyl]−7-nitro-2,1,3-benzoxadiazole) and three derivatives tested.

Metronidazole, amoxicillin, levofloxacin, and rifampicin were purchased from Sigma-Aldrich (Saint-Quentin-Fallavier, FR) and clarithromycin from Abcam (Amsterdam, NL). *Hp*-fld inhibitors (compounds IV, IVa, IVj, and IVk; Fig. 1) were synthesized as described (19). Stock solutions of antibiotics and compounds were prepared in dimethyl sulfoxide (DMSO) except for levofloxacin, which was dissolved in pure water and sodium hydroxide.

### Bacterial strains

The three *H. pylori* strains used in this study were ATCC 700392 (also named 26695), ATCC 43504, and Sydney Strain 1 (SS1). For each experiment, a frozen stock of *Hp* strains was streaked onto Columbia agar + 5% sheep blood (Bio-Rad, Marnes-la-Coquette, FR) and incubated for 3 days at 37°C in a microaerobic chamber (M35, Don Whitley Scientific, Bingley, UK) with 85% N_2_, 10% CO_2_, and 5% O_2_. Bacterial suspension of *Hp* was prepared from spiked colonies in Brain Heart Infusion (BHI) broth (Sigma-Aldrich, Saint-Quentin-Fallavier, FR) supplemented with 4% heat-inactivated fetal bovine serum (FBS) (Eurobio Scientific, Les Ulis, FR) and cultured under the same microaerophilic conditions at 37°C.

### MIC determination

Minimal inhibitory concentrations (MIC) of four *Hp*-fld inhibitors (compounds IV, IVa, IVj, and IVk), clarithromycin, metronidazole, amoxicillin, levofloxacin, and rifampicin were determined in duplicate or triplicate by the broth microdilution method for the three *Hp* strains, as previously described (19). Briefly, an inoculum of 10^6^ CFU/mL was incubated in BHI supplemented with FBS (BHI-FBS) under microaerophilic conditions at 37°C for 72 h, with concentrations of drugs ranging from 0.032 to 64 µg/mL. The MIC was determined after the addition of 5 µL of resazurin (0.2 mg/mL; Sigma-Aldrich, Saint-Quentin-Fallavier, FR), followed by an incubation of 2 h. Fluorescence measurements were performed with a FLUOstar Omega (BMG Labtech, Champigny-sur-Marne, FR) using excitation wavelengths of 480 nm and recording emission at 590 nm. The MIC was determined to be the lowest concentration, leading to a 70% decrease in fluorescence in comparison with the drug-free control.

### Checkerboard assays

Pairwise interactions between compound IV and other antibiotics (clarithromycin, metronidazole, amoxicillin, levofloxacin, or rifampicin) were studied on the three *Hp* strains using the method described by Salillas *et al*. (20). Briefly, a bacterial suspension, prepared in BHI-FBS to obtain a final bacterial inoculum of 2 × 10^6^ CFU/mL, was added to a 96-well plate containing serial twofold dilutions of compound IV along the abscissa and serial twofold dilutions of other antibiotics along the ordinate. After 72 h of incubation under microaerophilic conditions, the MIC of drugs alone or in combination was determined by fluorescence after the addition of resazurin (as described above for MIC determination). For each pair of molecules, the Fractional Inhibitory Concentration Index (FICI) was determined. The FICI represents the sum of the FICs of each drug tested, where the FIC is determined for each drug by dividing the MIC of the drug when used in combination with the MIC of the same drug when used alone. The FICI value can suggest a synergistic effect (FICI ≤ 0.5), an absence of interaction (0.5 < FICI ≤ 4), or an antagonist effect (FICI > 4) (21). Checkerboard assays were performed in triplicate for each tested condition (a pair of drugs and a bacterial strain).

### Time-kill studies

Time-kill studies (TKS) were performed with the *Hp* ATCC 700392 (26695) strain. Two hundred milliliters of a bacterial suspension containing approximately 10^6^ CFU/mL was prepared in a flask. After 16 h of incubation at 37°C in microaerophilic conditions under agitation (150 rpm), the suspension contained approximately 10^8^ CFU/mL. Then, 10 mL was transferred to individual tubes to be exposed from time 0 to 72 h to compound IV and/or another antibiotic twice and four times their MIC for the strain. The incubation was continued under the same conditions. At 0, 3, 6, 24, 48, and 72 h, 500 µL was sampled from each tube, and serial 10-fold dilutions were plated on Columbia agar + 5% sheep blood. After 3 days of incubation, the colonies on the agar were counted. TKS was performed at least in triplicate for each condition. The limit of quantification was 2 log_10_ CFU/mL, and, below this limit at 72 h, the bacteria were considered eradicated.

### PCR amplification and sequencing of the fldA gene

DNA was extracted by boiling from bacterial samples collected before (time 0) and after (time 72 h) exposure to compound IV at 2 and 4 µg/mL in three replicates of TKS. The *Hp* flavodoxin coding gene fldA was then amplified by PCR, according to the Phusion High-Fidelity DNA polymerase supplier recommendations (New England Biolabs) using the primers fldA F-gaaacgatattcgcgcaaaatgg and fldA R-atggtttcatcgctcaccaaa and 2 µL of *Hp* DNA. PCR products were purified and sequenced with fldA F primer provided by Eurofins Genomics (Ebersberg, Germany). Nucleotide sequences were compared using the BLASTN program of the National Center for Biotechnology Information (http://www.ncbi.nlm.nih.gov) and clustal Omega software at the EMBL-EBI online server (https://www.ebi.ac.uk/services).

## RESULTS

### Antibacterial activity of drugs alone

The MICs of four *Hp*-fld inhibitors related to compound IV (Fig. 1) and of clarithromycin, metronidazole, amoxicillin, levofloxacin, and rifampicin for three *Hp* strains [ATCC 700392 (26695), ATCC 43504, and Sydney Strain 1 (SS1)] are reported in Tables 1 and 2. The four *Hp*-fld inhibitors had MICs ranging from 0.5 to 64 µg/mL. Compounds IV and IVj had a higher antibacterial activity with MICs < 4 µg/mL than compounds IVa (MICs = 8 µg/mL) and IVk (16 < MICs < 64 µg/mL) (Table 1).

**TABLE 1:** Minimal inhibitory concentration (MIC) (in μg/mL) of compound IV and three of its derivatives (IVa, IVj, and IVk) for three Hp strains^*a*^

	MIC (µg/mL)			
	IV	IVa	IVj	IVk
ATCC 700392 (26695)	1	8	2–4	32
ATCC 43504	4	8	2–4	32–64
SS1	0.5	8	0.5–1	16

^*a*^ The values were obtained in duplicate.

**TABLE 2:** Minimal inhibitory concentration (MIC) (in μg/mL) of clarithromycin (Cla), metronidazole (Mnz), amoxicillin (Amx), levofloxacin (Lev), and rifampicin (Rif) for the three *Hp* strains^*a*^

	MIC (µg/mL)				
	Cla	Mnz	Amx	Lev	Rif
ATCC 700392(26695)	0.016	1	0.064	0.25	1
ATCC 43504	0.512	4	0.032	0.5	1
SS1	0.004	0.5	0.25	0.25	0.25

^*a*^ The values correspond to the mode of triplicates.

For the ATCC 700392, the MICs of clarithromycin, metronidazole, amoxicillin, levofloxacin, and rifampicin were below or equal to their respective clinical breakpoints for resistance of 0.25, 8, 0.125, 1, and 1 µg/mL (Table 2) (22). Only the MIC of clarithromycin for strain ATCC 43504 and the MIC of amoxicillin for strain SS1 were above the clinical breakpoints for resistance.

### Assessment of the interaction between compound IV and other antibiotics by checkerboard assays

The type of interaction between compound IV, the *Hp*-fld inhibitor with the lowest MIC on the three strains, and the other antibiotics was assessed by checkerboard assays. The FICIs for each pairwise comparison are provided in Table 3.

**TABLE 3:** Fractional inhibitory concentration index (FICI) of compound IV in combination with clarithromycin (Cla), metronidazole (Mnz), amoxicillin (Amx), levofloxacin (Lev), and rifampicin (Rif) for the three *Hp* strains

	FICI				
	Cla	Mnz	Amx	Lev	Rif
ATCC 700392(26695)	0.63	0.75	0.75	0.75	0.75
ATCC 43504	2	2	2	1	0.63
SS1	1	1	1	2	0.74

No synergistic effect (FICI ≤ 0.5) or antagonist effect (FICI > 4) was observed by combining compound IV with clarithromycin, metronidazole, amoxicillin, levofloxacin, or rifampicin, suggesting an indifference between molecules. For the three strains, the combination of compound IV with rifampicin led to a FICI lower than one, while for combinations with other antibiotics (clarithromycin, metronidazole, amoxicillin, and levofloxacin), a FICI lower than one was only observed for the ATCC 700392 strain.

### Time-kill studies to assess the time- and concentration-dependent antibacterial effects of drugs alone and in combination

The antimicrobial activity of compound IV, clarithromycin, metronidazole, amoxicillin, levofloxacin, and rifampicin alone and in combination was further characterized on *Hp* ATCC 700392 by performing time-kill studies on high bacterial inoculums over 72 h. The tested concentrations were equal to twice or four times the MIC of each molecule for the strain.

**FIG 2:**
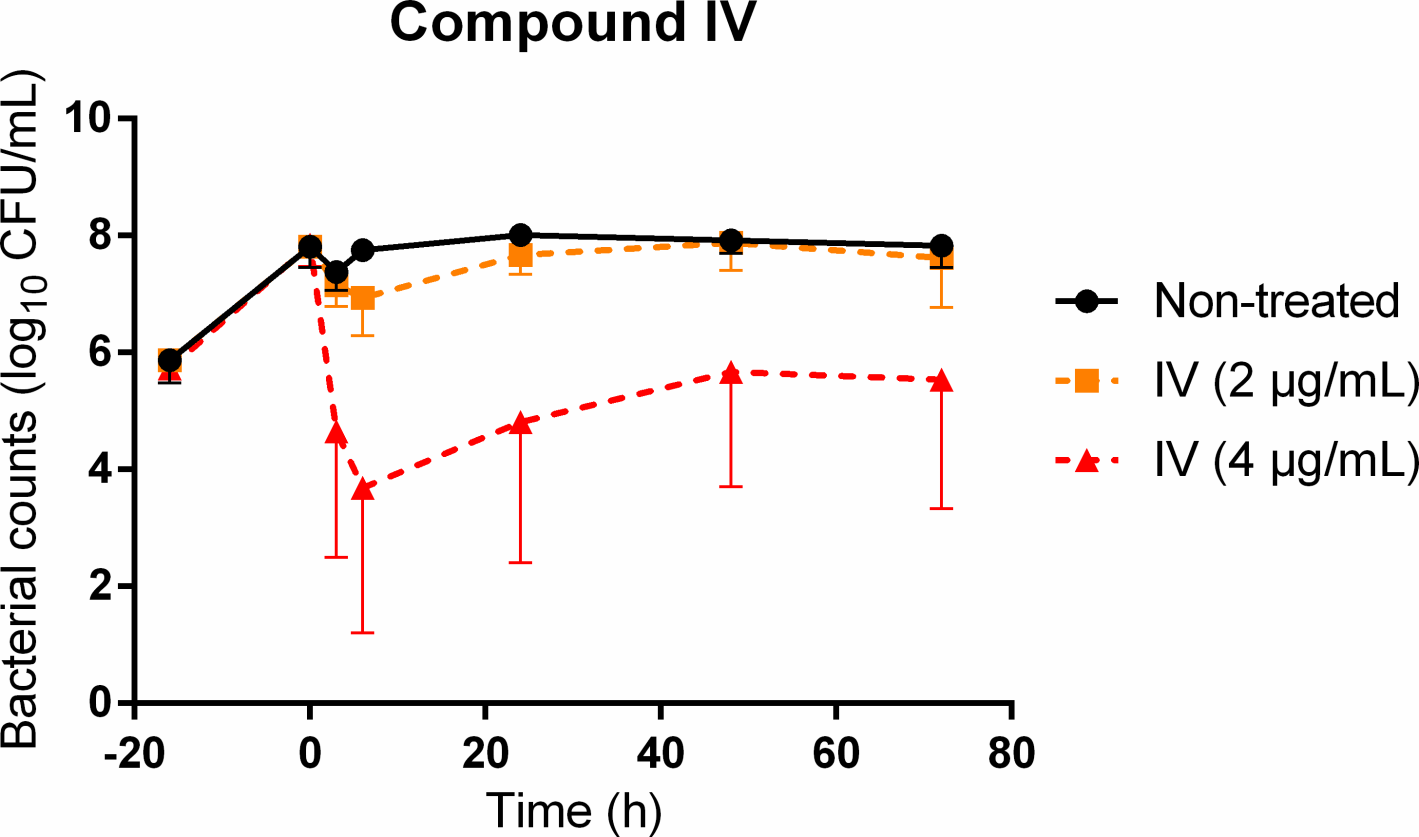
Observed viable counts of *Hp* ATCC 700392 following exposure to the *Hp*-fld inhibitor, compound IV. Control curves (black) and compound IV at twice the MIC (2 µg/mL) (orange curves) and at four times the MIC (4 µg/mL) (red curves) are shown. The bacteria were grown for 16 h without drugs before the addition of compound IV at time 0. Error bars represent the standard errors of the mean for eight independent biological replicates.

The bacterial counts at the time of drug addition were around 8 log_10_ CFU/mL. In control experiments without drugs, the bacterial counts remained constant over 72 h (except for a slight decrease observed at 3 h after the transfer from the flask to the individual tubes). For compound IV alone, the killing of bacteria was very mild at twice the MIC (2 µg/mL) with an average reduction of less than 1 log_10_ CFU/mL. At four times the MIC (4 µg/mL), a bactericidal effect corresponding to a killing of more than 3 log_10_ CFU/mL was obtained within 3–6 h, which was then followed by a slight regrowth (Fig. 2). Among the eight replicates at four times the MIC, the observed antibacterial effects were highly variable. For three of them, the initial decrease was less than 2 log_10_ CFU/mL, while for the other 5, there was a dramatic and fast decrease during the first 6 h leading to bacterial counts lower than the limit of quantification of 2 log_10_ CFU/mL, which was then followed by a slight regrowth to 4 log_10_ CFU/mL at 72 h (Fig. S1). No mutations in the fldA gene were detected in the bacterial populations that had regrown following exposure to 2 and 4 µg/mL of compound IV.

**FIG 3:**
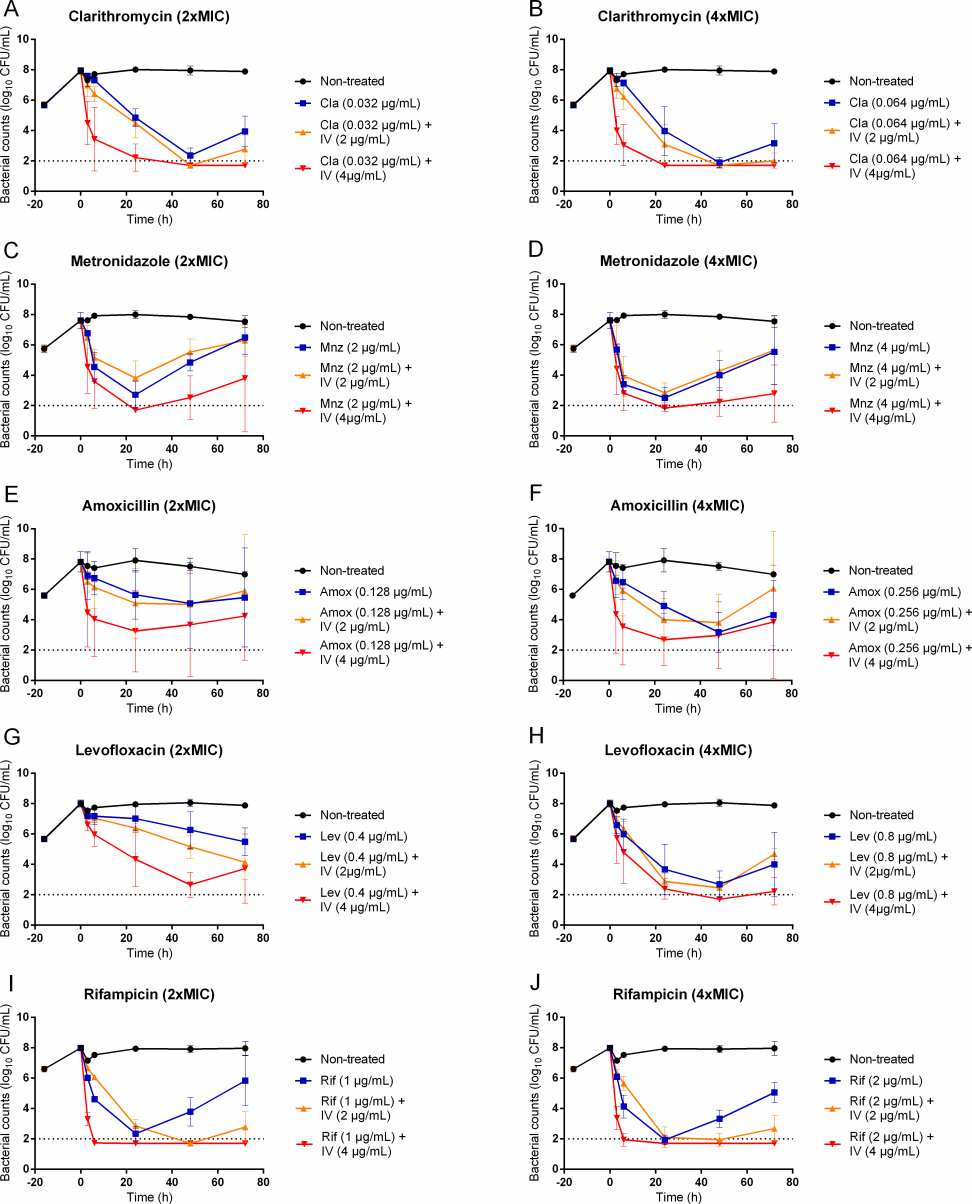
Observed viable counts of *Hp* ATCC 700392 following exposure to clarithromycin (**A and B**), metronidazole (**C and D**), amoxicillin (**E and F**), levofloxacin (**G and H**), and rifampicin (**I and J**), alone (blue curves) or in combination with the *Hp*-fld inhibitor, compound IV (IV), at twice (orange curves) or four times (red curves) the MIC. The bacteria were grown for 16 h without drugs before the addition of antibiotics at time 0. The horizontal dashed line corresponds to the limit of quantification (LOQ) in the assay (100 CFU/mL). Error bars represent the standard errors of the mean for three independent biological replicates.

For clarithromycin, metronidazole, amoxicillin, levofloxacin, and rifampicin, different antibacterial effects were observed over time depending on the drug (Fig. 3). A slight killing was observed over 72 h for levofloxacin and amoxicillin at twice the MIC (0.4 µg/mL and 0.128 µg/mL, respectively), with an average reduction of the initial inoculum of less than 3 log_10_ CFU/mL. Clarithromycin, metronidazole, and rifampicin (at both concentrations), and the highest concentrations of levofloxacin and amoxicillin led to an initial bactericidal effect with a reduction of more than 3 log_10_ CFU/mL over 24–48 h, which was frequently followed by regrowth. With antibiotics alone, eradication was only observed in one replicate out of three with both concentrations of amoxicillin and the highest concentration of clarithromycin and levofloxacin (Table 4). Despite the frequent regrowth, the final bacterial counts after 72 h of exposure to clarithromycin, metronidazole, amoxicillin, levofloxacin, and rifampicin at twice or four times the MIC remained lower than in control experiments on average.

**TABLE 4:** Number of replicates with eradication (bacterial counts below the limit of quantification) after 72 h of exposure to conventional antibiotics and/or compound IV

	Alone	+ Compound IV 2× MIC	+ Compound IV 4× MIC
No antibiotic		0/8	0/8
Clarithromycin			
2× MIC	0/3	1/3	3/3
4× MIC	1/3	2/3	3/3
Metronidazole			
2× MIC	0/3	0/3	1/3
4× MIC	0/3	0/3	2/3
Amoxicillin			
2× MIC	1/3	1/3	1/3
4× MIC	1/3	1/3	2/3
Levofloxacin			
2× MIC	0/3	0/3	1/3
4× MIC	1/3	0/3	2/3
Rifampicin			
2× MIC	0/3	1/3	3/3
4× MIC	0/3	1/3	3/3

The addition of compound IV at 2 µg/mL (corresponding to twice the MIC for the strain) to the conventional antibiotics at twice or four times the MIC led to similar time-kill curves as the antibiotics alone. The exception is rifampicin, for which the regrowth was slowed by the addition of compound IV. However, the addition of compound IV at 4 µg/mL (corresponding to four times the MIC for the strain) to the conventional antibiotics led to faster bacterial killing, with average bacterial counts lower than with antibiotics alone from the first sampling point at 3 h to the end of the experiments at 72 h. A bactericidal activity with a reduction of at least 3 log_10_ CFU/mL was observed when compound IV was added to any antibiotic, and the regrowth was reduced compared to antibiotics alone. The number of replicates with bacterial counts below the LOQ at 72 h was more frequent with the combination (Table 4) than with conventional antibiotics or compound IV alone. When the highest concentrations of compound IV were combined with clarithromycin and rifampicin, the regrowth was systematically prevented, with bacterial counts remaining below the LOQ for 72 h.

## DISCUSSION

*H. pylori* infection is highly prevalent worldwide, and the increase in bacterial resistance of *Hp* to currently used antibiotics has led to a decrease in the eradication rates by conventional triple and quadruple therapies. Novel and more effective approaches targeting new molecular pathways in the bacteria are urgently needed against *Hp*. Among the different strategies, new compounds inhibiting essential *Hp* metabolic pathways have been proposed. We have developed small-molecule inhibitors to target the essential *Hp* flavodoxin (*Hp*-fld), which is absent in humans, thus making it a promising pharmacological target (17, 23). Several *Hp*-fld inhibitors have previously shown antibacterial activity *in vitro* at low concentrations and in a mouse *Hp* infection model (20). The next step is to assess whether the combination of *Hp*-fld inhibitors with current recommended therapies could increase the eradication rates of *Hp*, especially when resistant mutants impair the activity of conventional antibiotics. In this regard, the types of interactions between *Hp*-fld inhibitors and conventional antibiotics including clarithromycin, metronidazole, amoxicillin, levofloxacin, and rifampicin were first investigated in this study using checkerboard assays. The impact of combinations involving Hp-fld inhibitors on bacterial eradication was subsequently evaluated using high inoculums, which are more likely to include spontaneous mutants.

Among the tested *Hp*-fld inhibitors derived from the lead compound IV, we found higher susceptibility of the *Hp* strains to compounds IV and IV-j compared to compounds IV-a and IV-k. These results were in agreement with those previously obtained on the strain ATCC 700392 (26695) (20) and led us to conduct further experiments with compound IV. The interaction of compound IV with conventional antibiotics was first evaluated *in vitro* by the checkerboard assay. Compound IV did not exhibit synergy or antagonism with the tested antibiotics, suggesting an absence of interaction between the three strains, as previously demonstrated for the combination with metronidazole and clarithromycin on the ATCC 700392 strain (20). Since the tested antibiotics are part of the current recommendations for patients and the tested strain showed susceptibility to all of them, we anticipated that these conventional antibiotics alone would probably eliminate a low inoculum. This would hinder the identification of any potential benefits of combining them with Hp-fld inhibitors. Hence, we evaluated the impact of the combination on the bacterial population under conditions that might compromise the activity of antibiotics, specifically a high inoculum. Therefore, the extent and time course of the antibacterial effect of compound IV alone or in combination with conventional antibiotics were assessed using time-kill studies on a high initial inoculum of over 3 days. Compound IV alone, at twice the MIC, did not reduce the bacterial population. Unfortunately, the high initial inoculum tested in this study made it impossible to distinguish between no antibacterial activity and bacteriostatic activity at this concentration. However, at four times the MIC, compound IV alone demonstrated a very fast bactericidal activity, with an average reduction of 3 log_10_ CFU/mL in 3–6 h. The average rate of killing (corresponding to the slope of the curve) was far higher for compound IV at four times the MIC than for the conventional antibiotics also tested at four times their MIC. However, the bacterial counts after 6 h of exposure to compound IV ranged from below the LOQ to 7.1 log_10_ CFU/mL depending on the replicates, demonstrating a high variability of the bacterial killing at this concentration, which was not observed at twice the MIC. This high variability was also not observed with conventional antibiotics, indicating that it cannot be solely attributed to the experimental conditions. These results for compound IV suggest a shift from no activity to very fast killing at a concentration of around four times the MIC. The variability can be explained by very small differences in the concentrations of compound IV in the replicates or, more likely, by differences in the expression of genes and differences in the metabolic activities of the bacteria. Here, the bacteria were exposed to drugs at the end of the exponential phase of growth after 16 h of incubation, but slight differences in the growth phase (late exponential phase or stationary phase), which could lead to significant differences in metabolic activity, could not be completely avoided. Independent of the extent of the initial reduction of the inoculum, regrowth was observed between 6 and 24 h in all of the replicates exposed to four times the MIC of compound IV, while the bacterial counts remained on average 3 log_10_ CFU/mL lower than the control after 72 h of exposure. There are several hypotheses to explain this regrowth, including the instability of the drug at 37°C, binding to plastic, and a decrease in susceptibility. Similar to a previous study with 5 × 10^7^ CFU/mL of the same strain ATCC 700392, exposed to different concentrations of compound IV (20), we did not observe any mutations in the fldA gene. However, we cannot exclude that *Hp* susceptibility to compound IV can be slightly affected by efflux pumps that could be overexpressed during exposure (20).

Monotherapies with clarithromycin, metronidazole, amoxicillin, levofloxacin, or rifampicin at four times their MIC demonstrated, as for compound IV, a bactericidal activity at 24 h on *Hp* in accordance with previously published results (24 – 26). Here, by prolonging the experiments up to 72 h, we found that exposure to a single drug often resulted in a regrowth that could be observed after 48 or 72 h. The selection of spontaneous mutant or *de novo* mutations during antibiotic exposure may be responsible for this regrowth. Indeed, *Hp* can become resistant to these antibiotics through single mutations in chromosomal genes (27, 28). For each antibiotic, the frequency of mutants and the mutation rate per cell division ranged from 10^−8^ to 10^−10^, making it likely that mutants were present in the initial inocula of around 8 log_10_ CFU/mL used in this study before the addition of drugs. These random mutation resistance mechanisms support the recommendation of combined treatments, as the likelihood of a bacterium having two independent point mutations that make it resistant to two antibiotics is extremely low.

By combining one conventional antibiotic with compound IV at four times the MIC, bacterial killing was promoted with a faster bactericidal effect than antibiotics alone. This feature is promising since fast killing could be favorable to reduce the cell divisions and thus the risk of the emergence of *de novo* resistance. When compound IV was combined with clarithromycin or rifampicin at twice or four times the MIC, *Hp* became undetectable and did not regrow during the 72 h study, suggesting complete eradication. A regrowth was only observed for one replicate out of three for amoxicillin, levofloxacin, and metronidazole at four times the MIC at 72 h. These results suggest that the addition of compound IV to conventional antibiotics prevented the selection of resistant mutants during drug exposure. The tested initial inoculum for these time-kill studies was higher than the standard inoculum of 5 × 10^5^ CFU/mL which is classically used for MIC determination, and the probability of the presence of a spontaneously resistant mutant before drug exposure was high. The eradication of bacteria suggests that compound IV killed both antibiotic-resistant mutants present before exposure and those that could have emerged by mutation during drug exposure.

In conclusion, the prevention of regrowth by adding compound IV to amoxicillin, metronidazole, levofloxacin, and rifampicin is very promising for the patients infected by clarithromycin-resistant strains. Indeed, clarithromycin is often recommended in first-line nonbismuth quadruple therapy for the eradication of *Hp*, and its replacement remains challenging, leading to the presence of clarithromycin-resistant *Hp* in the WHO priority list (11). Adding *Hp*-fld inhibitors to these antibiotics could increase efficacy, lead to a higher eradication rate, and, more importantly, reduce the selection of resistance to these drugs. *Hp*-fld inhibitors are promising drug candidates to increase the eradication rates of *H. pylori*. However, additional studies are needed to further elucidate their efficacy and safety in animal models of infections and in patients.

## SUPPLEMENTAL MATERIAL

Figure S1 spectrum.02623-23-s0001.docx Observed viable counts of *Hp* ATCC 700392 following exposure to the *Hp*-fld inhibitor compound IV at four times the MIC.

ASM does not own the copyrights to Supplemental Material that may be linked to, or accessed through, an article. The authors have granted ASM a non-exclusive, world-wide license to publish the Supplemental Material files. Please contact the corresponding author directly for reuse.
