# Supplementary material for: Antimicrobial combinations against Helicobacter pylori including benzoxadiazol-based flavodoxin inhibitors: in vitro characterization
Source: Microbiol Spectr. 2023 Dec 12;12(1):e02623-23. doi: 10.1128/spectrum.02623-23 (PMC10783109; doi:10.1128/spectrum.02623-23)
Supplement: Figure S1 — Observed viable counts of Hp ATCC 700392 following exposure to the Hp-fld inhibitor compound IV at four times the MIC. [file spectrum.02623-23-s0001.docx]

**Figure S1:** Observed viable counts of *Hp* ATCC 700392 following exposure to the *Hp*-fld inhibitor, compound IV at four times the MIC (4 µg/mL) are shown. The mean and standard errors of the 8 replicates are represented with a full line. Among these replicates, three of them led to a low antibacterial effect and five of them led to high antibacterial effects (dashed curves). The bacteria were grown for 16 h without drugs before the addition of antibiotics at time 0. Error bars represent the standard errors of the mean for independent biological replicates.
